# The efficacy and safety of sacubitril/valsartan compared with ACEI/ARB in the treatment of heart failure following acute myocardial infarction: a systematic review and meta-analysis of randomized controlled trials

**DOI:** 10.3389/fphar.2023.1237210

**Published:** 2023-08-04

**Authors:** Jinquan Gao, Xin Zhang, Mengzhuo Xu, Shisu Deng, Xiaoping Chen

**Affiliations:** ^1^ Department of Cardiology, West China Hospital, Sichuan University, Chengdu, China; ^2^ Chongzhou People’s Hospital, Chongzhou, China

**Keywords:** heart failure following acute myocardial infarction, sacubitril-valsartan, angiotensin-converting enzyme inhibitor, angiotensin receptor blocker, randomized controlled trial, meta-analysis

## Abstract

**Purpose:** To systematically assess the efficacy and safety of sacubitril/valsartan (SV) by comparison with angiotensin-converting enzyme inhibitors (ACEIs) or angiotensin receptor blockers (ARBs) for the treatment of heart failure caused by acute myocardial infarction (HF-AMI) based on current randomized controlled trials (RCTs).

**Methods:** Several electronic databases were searched up to 27 May 2023. Primary endpoints were the efficacy including the left ventricular ejection fraction (LVEF), left ventricular end-diastolic diameter (LVEDD), N-terminal pro-B type natriuretic peptide (NT-proBNP) and 6-min walk test (6MWT) and secondary endpoints were the safety including the major adverse cardiovascular event (MACE) and adverse reaction (AE).

**Results:** A total of 14 RCTs were included and all patients were from China. Among included 1,991 patients, 997 patients received SVs and 994 patients received ACEIs/ARBs. The pooled results demonstrated that patients in the SV group showed significantly better efficacy representing as increased LVEF [weighted mean difference (WMD): 4.43%, 95% confidence interval (CI): 2.84%–6.02%, *p* < 0.001] and 6MWT (WMD: 30.84 m, 95% CI: 25.65 m–36.03 m, *p* < 0.001) and decreased LVEDD (WMD: −3.24 mm, 95% CI: −4.96 mm ∼ -1.52 mm, *p* < 0.001) and NT-proBNP (WMD: −188.12 pg/mL, 95% CI: −246.75 pg/mL ∼ 129.49 pg/mL, *p* < 0.001), which was also verified by subgroup analysis based on the history of percutaneous coronary intervention (PCI). Besides, the SV group showed significantly lower incidence rate of MACE [relative risk (RR): 0.60, 95% CI: 0.47–0.75, *p* < 0.001] and patients receiving SVs in the non-PCI group also showed lower incidence of AE (RR: 0.38, 95% CI: 0.20–0.71, *p* = 0.002).

**Conclusion:** For the treatment of HF-AMI, SV is more effective and safer than ACEI/ARB based on current evidence, but more high-quality RCTs are still needed to verify above findings.

## 1 Introduction

Acute myocardial infarction (AMI) is myocardial necrosis caused by disruption of blood flow following rupture of unstable plaque in the coronary artery. It is the most common manifestation of coronary heart disease and a serious threat to human health (Fernandez Rico et al., 2022; Ciftci et al., 2023; Vergallo and Patrono, 2023). Despite great advances in medical care, AMI has long been the leading cause of disability and death worldwide (Vergallo and Patrono, 2023). In the United State, 1.5 million cases are reported each year (Khera et al., 2021; Berwanger et al., 2022). Meanwhile, in China, the incidence of AMI increased gradually from 2002 to 2018 (Li et al., 2015; Huo et al., 2019). Over the past decade, great advances in early revascularization and AMI management have results in a 95% of 30-day survival rate for AMI with ST segment elevation (Li et al., 2015). However, about 25% of new AMI patients will develop heart failure (HF) within 1 year and 75% of all patients will develop HF within 5 years (Yandrapalli et al., 2021; Solomonchuk et al., 2022). In the next few decades, it is speculated that the number of patients with HF after AMI (HF-AMI) will substantially increase because of the population growth, aging and comorbidities increase (Yandrapalli et al., 2021; Solomonchuk et al., 2022).

For the treatment of HF-AMI, angiotensin-converting enzyme inhibitors (ACEIs)/angiotensin receptor blockers (ARBs) has been the basic drugs for HF-AMI. However, in recent years, with more research on myocardial infarction, ventricular remodeling and HF, a variety of new drugs are emerging including the current landmark new drug sacubitril/valsartan (SV) (Hajra et al., 2019). A number of clinical trials have well indicated its efficacy in treating HF with reduced ejection fraction is significantly superior to ACEI, which has been recommended by several domestic and foreign guidelines (Abdin et al., 2022; Bhatt et al., 2022; Zeymer et al., 2022). In 2021, the European Heart Association recommended that SV could replace ACEI as the first choice for patients with acute or chronic HF with reduced ejection fraction, so as to reduce the risk of HF visits or death (Kapelios et al., 2019; Zeymer et al., 2022). The results of the PARAMOUNT-HF and PARADIGM-HF studies have both showed that SV significantly improved the indicators of cardiac function and ventricular remodeling in patients with HF compared with ACEI/ARB (Docherty et al., 2020), which is consistent with the results of PROVE-HF and EVALUATE-HF trials (Myhre et al., 2022). Although SV has shown obvious advantages in the treatment of HF, its efficacy and safety in patients with HF after AMI has not been fully determined.

Therefore, this study aimed to systematically identify the efficacy and safety of SV by comparison with ACEIs/ARBs for the treatment of HF-AMI based on current evidence provided by randomized controlled trials (RCTs).

## 2 Materials and methods

The current systematic review and meta-analysis was performed according to the Preferred Reporting Items for Systematic Review and Meta-Analyses 2020 (Page et al., 2021).

### 2.1 Literature search

The PubMed, EMBASE, Web of Science, Cochrane library and CNKI databased were searched from inception to 27 May 2023. The following terms were used during the research: sacubitril-valsartan, valsartan-sacubitril, entresto, LZC696, SV, myocardial infarction, cardiovascular stroke, myocardial infarct, heart attack, heart failure, cardiac failure, randomized controlled trial and RCT. Detailed search strategy was as follows: (sacubitril-valsartan OR valsartan-sacubitril OR entresto OR LCZ696 OR SV) AND (myocardial infarction OR cardiovascular stroke OR myocardial infarct OR heart attack) AND (heart failure OR cardiac failure) AND (randomized controlled trial OR RCT).

### 2.2 Inclusion criteria

The inclusion criteria were as follows: 1) patients were diagnosed with HF-AMI according to latest guidelines and expert consensuses (Branch of Cardiovascular Physicians CMDACCHATECWGotPaToHFAMI, 2020; Jenča et al., 2021); 2) patients were randomized to receive the SVs or ACEIs/ARBs for at least 1 month and all patients received same basic therapies including the antiplatelet, lipid-lowering and beta-blocker treatment; 3) randomized controlled trials (RCTs) which enrolled 100 or more participants; 4) at least one of following outcomes was compared between the SV and ACEI/ARB groups: LVEF, LVEDD, NT-proBNP, 6MWT, MACE and AR; 5) full texts were available and enough data were provided for the calculation of weighted mean difference (WMD) or (and) relative risk (RR) with corresponding 95% confidence interval.

### 2.3 Exclusion criteria

The following criteria were applied: 1) studies with a small sample size (<100 participants); 2) insufficient or duplicated data; 3) conference abstracts, letters, editorials, case reports or reviews.

### 2.4 Data collection

The following information was retracted from included studies: the name of first author, publication year, country, sample size, history of percutaneous coronary intervention (PCI), drugs of control group and follow-up time. Primary endpoints were the efficacy including the LVEF, LVEDD, NT-proBNP and 6MWT and secondary endpoints were the safety including the MACE and AE, respectively.

### 2.5 Methodological quality assessment

The quality of all included RCTs was evaluated according to the modified Jadad Scale score tool which consisted of the randomization, concealment of allocation, double blinding and withdrawals and dropouts (Clark et al., 1999; Bhandari et al., 2001). RCTs with a modified Jadad Scale of one to three and four to seven were defined as low-quality and high-quality studies, respectively (Clark et al., 1999; Bhandari et al., 2001).

### 2.6 Statistical analysis

RevMan 5.3 and STATA 12.0 software were applied for the analysis. Continuous variables including the LVEF, LVEDD, NT-proBNP and 6MWT after the SV or ACEI/ARB treatment were compared and analyzed using the WMD and corresponding 95% CI. Binary variables including the MACE and AR were compared using the RR with 95% CI. The heterogeneity between studies was assessed using I^2^ statistics and the Q test. If significant heterogeneity was observed representing as I^2^ > 50% and/or *p* < 0.1, the random effects model was applied; otherwise, the fixed effects model was used. Besides, subgroup analysis based on the history of PCI was conducted. Sensitivity analysis was conducted to detect the sources of heterogeneity and assess the stability of the overall results. Furthermore, Begg’s funnel plot and Egger’s test were conducted to detect publication bias, and significant publication bias was defined as *p* < 0.05 (Begg and Mazumdar, 1994; Egger et al., 1997). If significant publication bias was observed, then the trim-and-fill method was applied to detect potentially unpublished publications (Shi and Lin, 2019).

## 3 Results

### 3.1 Literature search and selection

**FIGURE 1:**
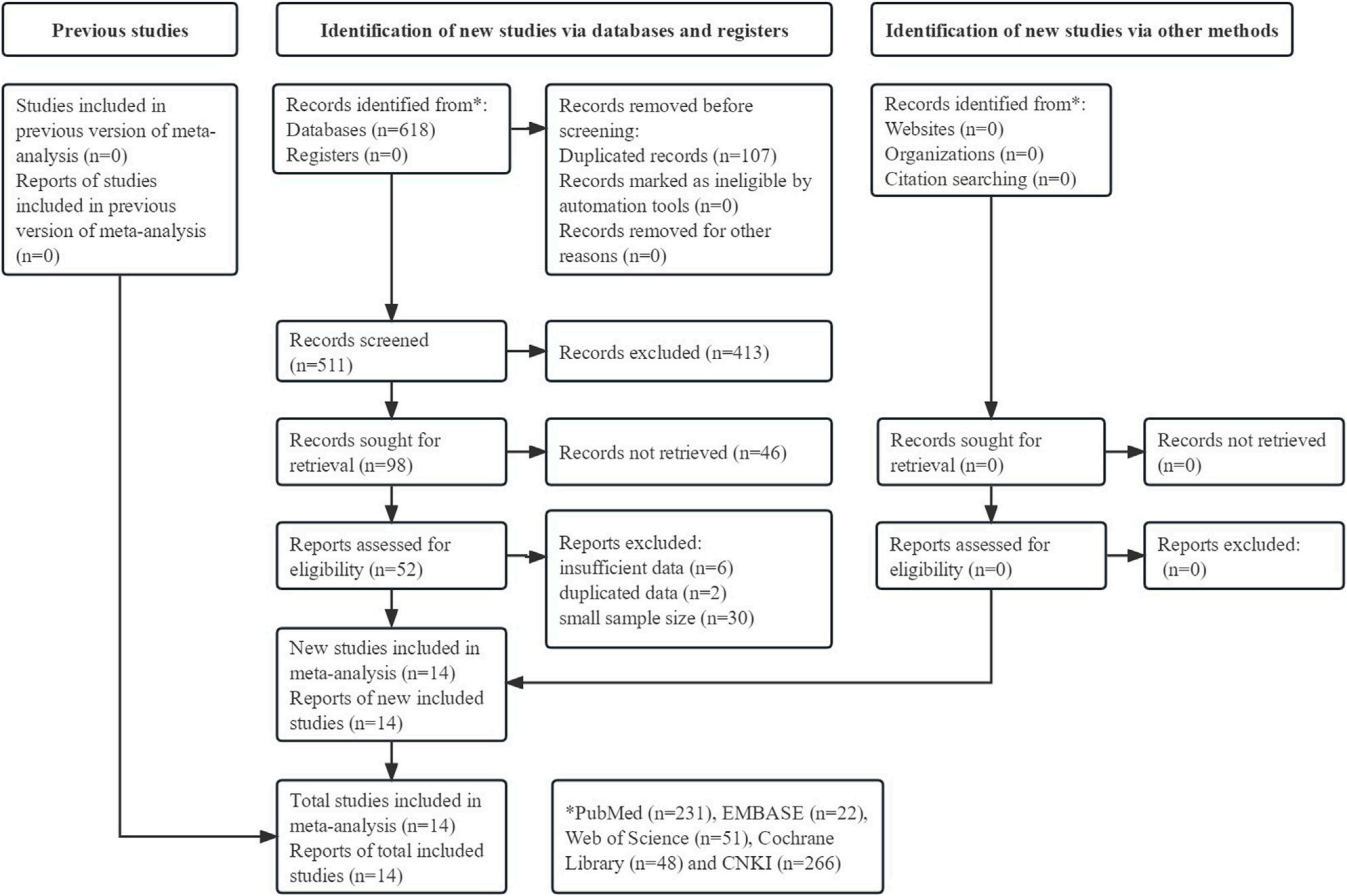
Prisma flow diagram of this meta-analysis.

Initially, 618 records were identified from the five databases and 107 duplicated records were directly removed. After reviewing the titles and abstracts, 413 and 46 records were excluded, respectively. Then, 38 publications were further excluded after reviewing the full texts. Eventually, 14 RCTs were included in this meta-analysis (Cao and Zhao, 2020; Wang et al., 2020; Zhao, 2020; Cui, 2021; Dong, 2021; Haiyan and Xianghua, 2021; Jiang, 2021; Liu and Zhou, 2021; Fu and Xu, 2022; Sheng, 2022; Wang et al., 2022; Xie, 2022; Zhou, 2022; Jiao et al., 2023). (Figure 1)

### 3.2 Basic characteristics of included studies

All included studies were from China with the overall sample size of 1,991 participants. Among these 1,991 patients, 997 and 994 patients were randomized to the SV and ACEI/ARB groups, respectively. Nine (Wang et al., 2020; Cui, 2021; Dong, 2021; Haiyan and Xianghua, 2021; Liu and Zhou, 2021; Fu and Xu, 2022; Wang et al., 2022; Zhou, 2022) and five (Cao and Zhao, 2020; Zhao, 2020; Jiang, 2021; Xie, 2022; Jiao et al., 2023) RCTs were separately defined as high-quality and low-quality studies according to modified Jadad Scale. Specific information was shown in Table 1.

**TABLE 1:** Basic characteristics of included studies.

Author	Year	Country	Sample size (EG)	Sample size (CG)	PCI	Control intervention	Endpoints	Follow-up time (months)	Modified jadad scale
Cao and Zhao (2020)	2020	China	68	68	No	NR	①②④⑥	2	3
Wang et al. (2020)	2020	China	80	80	Yes	Valsartan	①③⑥	6	4
Zhao (2020)	2020	China	62	61	Yes	Valsartan	①③	6	3
Haiyan and Xianghua (2021)	2021	China	68	69	Yes	Enalapril	①③⑤⑥	6	4
Cui (2021)	2021	China	104	98	Yes	Valsartan	①②③④⑤⑥	6	4
Dong (2021)	2021	China	64	64	Yes	Enalapril	①③⑤⑥	6	4
Jiang (2021)	2021	China	100	100	No	Valsartan	①②③④⑥	2	3
Liu and Zhou (2021)	2021	China	60	60	Mixed	Benazepril	①②③⑥	2	4
Wang et al. (2022)	2022	China	60	60	No	ACEI	⑥	1	4
Fu and Xu (2022)	2022	China	63	63	Yes	Fosinopril sodium	①②③⑤⑥	6	4
Sheng (2022)	2022	China	60	60	Yes	Benazepril	①②③⑤⑥	6	4
Xie (2022)	2022	China	50	50	No	Valsartan	①②	NR	3
Zhou (2022)	2022	China	107	110	Yes	Benazepril	①②③⑤⑥	6	5
Jiao et al. (2023)	2023	China	51	51	Yes	Enalapril	①②③⑥	6	3

EG: experimental group; CG: control group; PCI: percutaneous coronary intervention; NR: not reported; ACEI: angiotensin-converting enzyme inhibitor; ①: LVEF: left ventricular ejection fraction; ②: LVEDD: left ventricular end-diastolic diameter; ③: NT-proBNP: N-terminal pro-B, type natriuretic peptide; ④: 6-min walk test; ⑤: major adverse cardiovascular event; ⑥: adverse reaction.

### 3.3 Efficacy of SV by comparison of ACEI/ARB for HF-AMI

**FIGURE 2:**
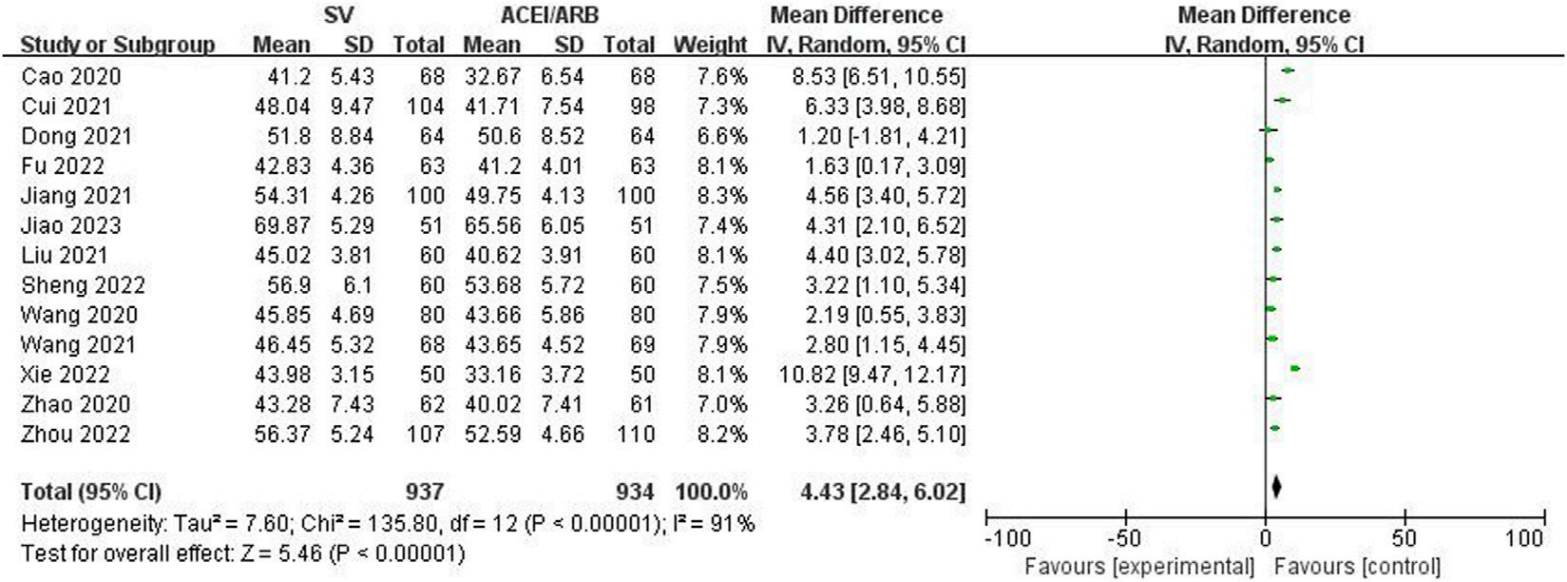
Forest plot for the left ventricular ejection fraction in two groups.

**FIGURE 3:**
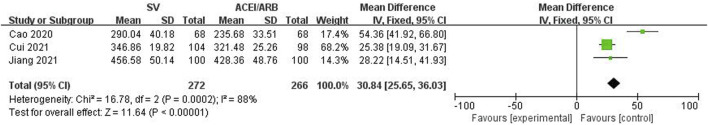
Forest plot for the 6-min walk test in two groups.

**FIGURE 4:**
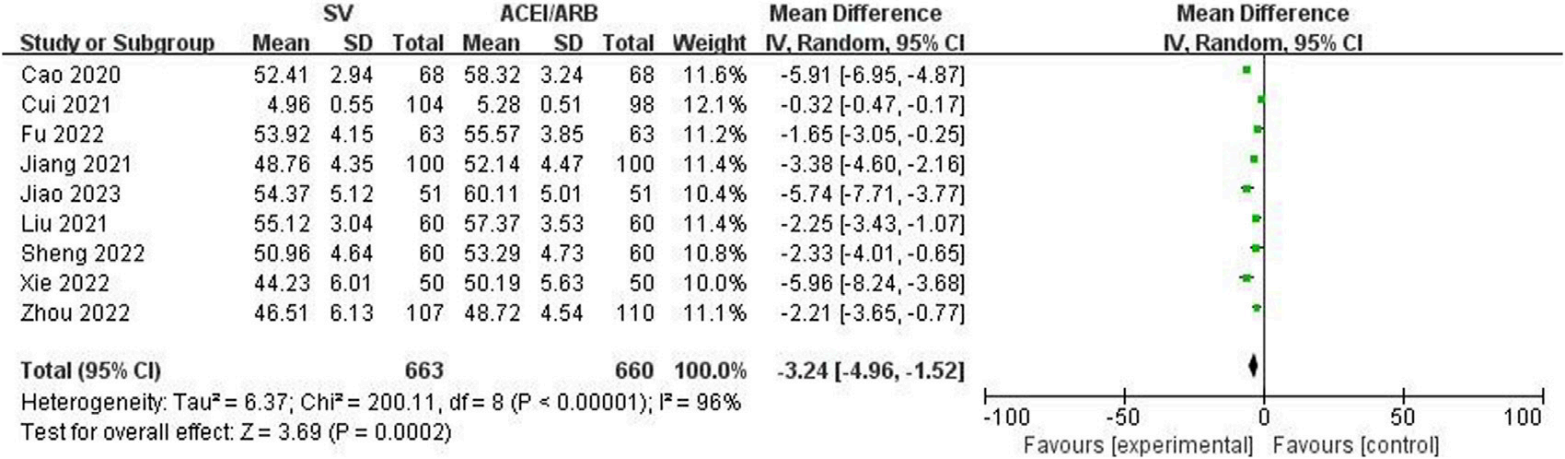
Forest plot for the left ventricular end-diastolic diameter in two groups.

**FIGURE 5:**
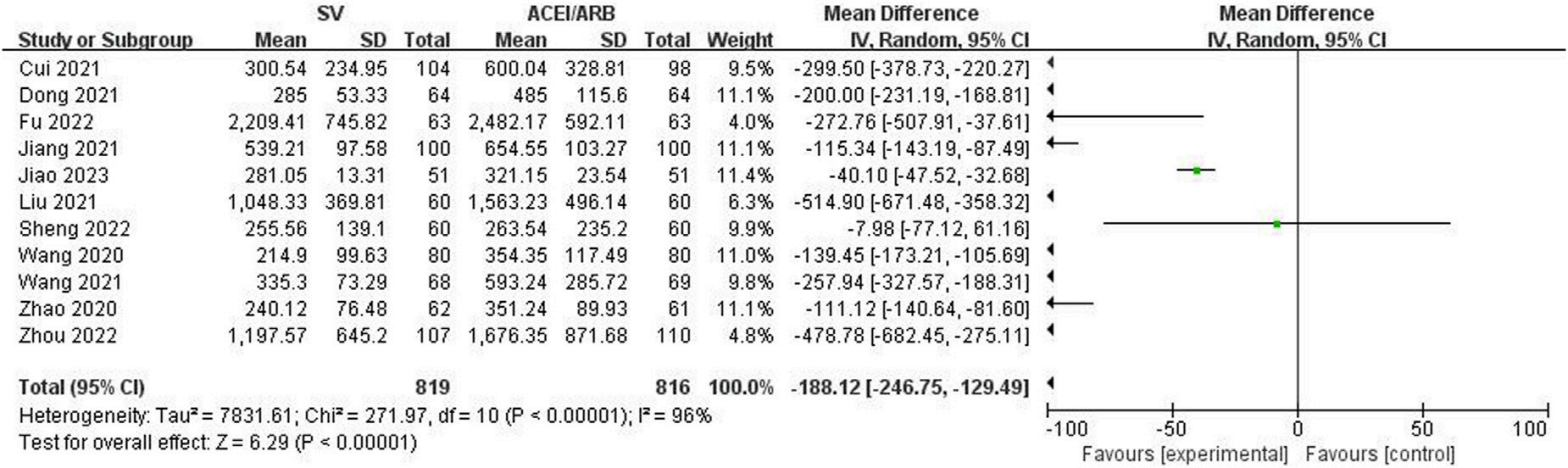
Forest plot for the NT-proBNP N-terminal pro-B type natriuretic peptide in two groups.

**FIGURE 6:**
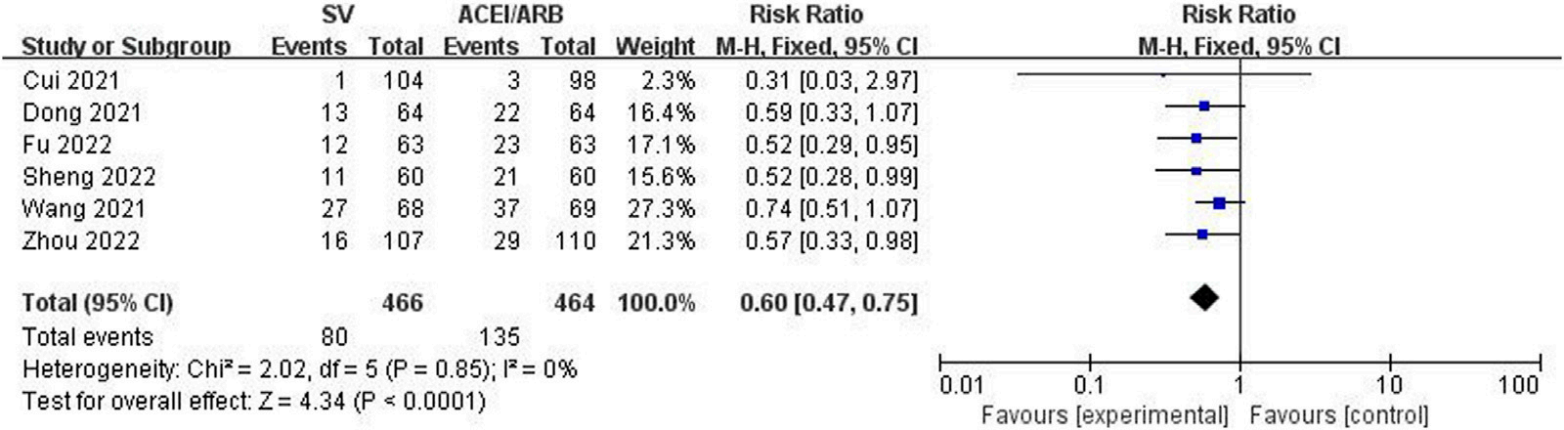
Forest plot for the major adverse cardiovascular event in two groups.

The LVEF, LVEDD, NT-proBNP and 6MWT values after the SV or ACEI/ARB treatment were pooled to identify the efficacy of SV in the treatment of HF-AMI compared to ACEI/ARB. The pooled results demonstrated that patients in the SV group showed significantly better efficacy representing as increased LVEF (WMD: 4.43%, 95% CI: 2.84%–6.02%, *p* < 0.001; I^2^: 91%, *p* < 0.001) (Figure 2) and 6MWT (WMD: 30.84 m, 95% CI: 25.65 m–36.03m, *p* < 0.001; I^2^: 88%, *p* < 0.001) (Figure 3) and decreased LVEDD (WMD: −3.24 mm, 95% CI: −4.96 mm ∼ -1.52 mm, *p* < 0.001; I^2^: 96%, *p* < 0.001) (Figure 4) and NT-proBNP (WMD: −188.12 pg/mL, 95% CI: −246.75 pg/mL ∼ -129.49 pg/mL, *p* < 0.001; I^2^: 96%, *p* < 0.001) (Figure 5).

Furthermore, subgroup analysis based on the history of PCI manifested similar results. Patients with and without the history of PCI in the SV group both showed increased LVEF (WMD: 3.31%, 95% CI: 2.46%–4.16%, *p* < 0.001; WMD: 7.95%, 95% CI: 3.79%–12.12%, *p* < 0.001) and 6MWT (WMD: 25.38 m, 95% CI: 19.09 m–31.67 m, *p* < 0.001; WMD: 41.46 m, 95% CI: 15.84 m–67.07 m, *p* = 0.002) and decreased LVEDD (WMD: 2.28 mm, 95% CI: −3.73 mm ∼ -0.84 mm, *p* = 0.002; WMD: −5.01 mm, 95% CI: −6.87 mm ∼ -3.15 mm, *p* < 0.001) and NT-proBNP (WMD: −201.62 pg/mL, 95% CI: −269.92 pg/mL ∼ -133.32 pg/mL, *p* < 0.001; WMD: −115.34 pg/mL, 95% CI: −143.19 pg/mL ∼ -87.49 pg/mL, *p* < 0.001). Detailed data were presented in Table 2.

**TABLE 2:** Results of meta-analysis.

	No. studies	WMD/RR	95% confidence interval	*p*-value	I^2^	*p*-value
Left ventricular ejection fraction	13	4.43%	2.84%–6.02%	<0.001	91%	<0.001
PCI group	10	3.31%	2.46%–4.16%	<0.001	53%	0.02
Non-PCI group	3	7.95%	3.79%–12.12%	<0.001	96%	<0.001
Left ventricular end-diastolic diameter	9	−3.24 mm	−4.96 mm ∼ -1.52 mm	<0.001	96%	<0.001
PCI group	6	−2.28 mm	−3.73 mm ∼ -0.84 mm	0.002	91%	<0.001
Non-PCI group	3	−5.01 mm	−6.87 mm ∼ -3.15 mm	<0.001	81%	0.006
N-terminal pro-B type natriuretic peptide	11	−188.12 pg/mL	−246.75 pg/mL ∼ -129.49 pg/mL	<0.001	96%	<0.001
PCI group	10	−201.62 pg/mL	−269.92 pg/mL ∼ -133.32 pg/mL	<0.001	97%	<0.001
Non-PCI group	1	−115.34 pg/mL	−143.19 pg/mL ∼ -87.49 pg/mL	<0.001	-	-
6-min walk test	3	30.84 m	25.65m–36.03 m	<0.001	88%	<0.001
PCI group	1	25.38 m	19.09m–31.67 m	<0.001	-	-
Non-PCI group	2	41.46 m	15.84m–67.07 m	0.002	87%	0.006
Major adverse cardiovascular events	6	0.60	0.47–0.75	<0.001	0%	0.85
PCI group	6	0.60	0.47–0.75	<0.001	0%	0.85
Adverse reaction	12	0.74	0.51–1.09	0.13	54%	0.01
PCI group	9	0.89	0.60–1.32	0.57	48%	0.05
Non-PCI group	3	0.38	0.20–0.71	0.002	0%	0.40

PCI: percutaneous coronary intervention; WMD: weighted mean difference; RR: relative risk.

### 3.4 Safety of SV by comparison of ACEI/ARB for HF-AMI

**FIGURE 7:**
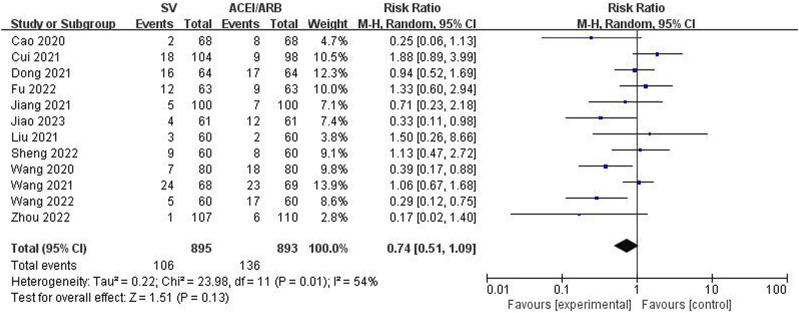
Forest plot for the adverse reaction in two groups.

The incidence rates of MACE and AR were compared between two groups to identify the safety of SV in the treatment of HF-AMI compared to ACEI/ARB. The pooled results revealed that SV group showed significantly lower incidence rate of MACE (RR: 0.60, 95% CI: 0.47–0.75, *p* < 0.001; I^2^: 0%, *p* = 0.85) (Figure 6). Meanwhile, there was no significant difference in the incidence of AE between the two groups (RR: 0.74, 95% CI: 0.51–1.09, *p* = 0.13; I^2^: 54%, *p* = 0.01) (Figure 7).

However, patients without the history of PCI in the SV group showed significantly decreased risk of AE (RR: 0.38, 95% CI: 0.20–0.71, *p* = 0.002; I^2^: 0%, *p* = 0.40) (Table 2).

### 3.5 Sensitivity analysis and publication bias

Sensitivity analysis for the LVEF, LVEDD and NT-proBNP were performed, which indicated the stability of the results and none of included studies caused an obvious impact on the overall results (Supplementary Figure S1A–C).

Besides, asymmetric Begg’s funnel plots (Supplementary Figure S2A,B) and P values of Egger’s test (*p* = 0.004; *p* = 0.002) indicated obvious publication bias, but the results of trim-and-fill method showed that potentially unpublished publications did not cause a significant impact on the overall conclusion. The symmetric Begg’s funnel plot (Supplementary Figure S2C) and *p* = 0.625 of Egger’s test indicated nonsignificant publication bias for LVEDD.

## 4 Discussion

The current meta-analysis has demonstrated that SV is superior to ACEI/ARB in the treatment of HF-AMI based on current evidence by relevant RCTs. In detail, patients receiving SVs are more likely to experience significantly better efficacy representing as increased LVEF and 6MWT and decreased LVEDD and NT-proBNP. Furthermore, patients in the SV group are less like to have MACE and AE. However, due to the limitations existed in this meta-analysis and low quality of some included studies, more high-quality RCTs from other countries are still needed to further verify above findings.

SV is a dual inhibitor for angiotensin receptor and neprilysin and could simultaneously regulate the renin-angiotensin-aldosterone system (RAAS) and natriuretic peptide system (NPS) (Kario, 2018). The loss of myocardial cells, ventricular remodeling and activation of neuroendocrine system are the basic pathological processes of HF-AMI and the RAAS, sympathetic nervous system (SNS) and NPS play essential roles in this process (Singh et al., 2017; Pascual-Figal et al., 2021). LVEF, LVEDD and NT-proBNP are commonly used to assess the ventricular remodeling and NPS, and our results have indicated that SV could better improve ventricular remodeling and NPS. Overactivation of RAAS can lead to increased aldosterone secretion, vasoconstriction, hypertrophy and apoptosis of cardiomyocytes, resulting in water and sodium retention and myocardial fibrosis, thus triggering and aggravating symptoms of heart failure (Mochel et al., 2019; Wachter et al., 2020). Therefore, the inhibition of RAAS is vital for the treatment of patients of HF-AMI, which could be reached by both SV and ACEI/ARB. Besides, SV also enhances NPS by inhibiting neprilysin and NPS plays a strong role in anti-myocardial hypertrophy, anti-myocardial fibrosis and antagonistic overactivation of sympathetic and RAAS (Lillyblad, 2015; Yamamoto and Rakugi, 2021). Natriuretic peptides are commonly applied as markers for cardiovascular diseases including the HF in clinics. It has been reported that SV could produce a synergistic effect by reducing the angiotensin II-related signal transduction pathways and increasing the level of natriuretic peptides (Volpe et al., 2023). Above researches explain why the efficacy of SV is superior to ACEI/ARB. Besides, natriuretic peptides exert many cardiac beneficial effects such as the ability to protect cardiomyocytes by stimulating autophagy through the activation of transcription factor EB after myocardial and in HF with reduced ejection fraction (Forte et al., 2023; Raffa et al., 2023). Therefore, the results of this meta-analysis have well demonstrated that SV is more effective and safer than ACEI/ARB for the treatment of HF-AMI based on 14 relevant RCTs.

Our results have indicated that SV could significantly improve the ventricular remodeling and decrease the risk of MACE, which is inconsistent with the findings of Docherty et al. Docherty et al. (2021). In their RCT, SV did not significantly reduce LVEF, left ventricular end-systolic volume index (LVEVI) or NT-proBNP compared with valsartan (Docherty et al., 2021). The main reason may be the different enrolled patients. Although the included patients were all MI patients, Docherty et al. included patients with asymptomatic left ventricular systolic dysfunction or transient pulmonary congestion after MI, and the baseline level of NT-proBNP was low, which did not conform to HF diagnosis. Asymptomatic left ventricular systolic dysfunction after MI is an important risk factor for developing HF, which may increase the likelihood of developing HF in the future. However, this does not indicate that all patients will develop HF, and a considerable number of patients will return to normal. Ventricular remodeling and neuroendocrine system activation of patients in their study are relatively mild. There is no need to enhance NPS to antagonize the RAAS system, so it is only necessary to use ACEI/ARB to inhibit the RAAs system, and SV does not show an advantage. HF-AMI patients included in this meta-analysis showed HF-related symptoms and signs, high level of NT-proBNP, high degree of ventricular remodeling and overactivation of neuroendocrine system, and requires stronger NPS to antagonize it. Therefore, SV with enhanced effect of NPS showed better efficacy.

In this meta-analysis, we excluded 30 RCTs with small sample sizes (<100 cases) in order to improve the reliability of conclusions. These 30 studies were all published in Chinese with relatively low quality except the study by Docherty et al. Docherty et al. (2021). Their sample sizes ranged from 30 to 98 cases and were published between 2019 and 2023. After careful team discussion, we decided to exclude these studies to reduce the bias caused by small sample sizes, so as to make the conclusion more rigorous and reliable. Notably, some included studies did not report baseline values of efficacy endpoints, thus we compared the post-treatment values between the SV and ACEI/ARB groups instead of the changes of efficacy endpoints. Besides, we did not establish the inclusion criteria for Chinese populations initially, but all available studies focused on Chinese patients. Therefore, our findings about the efficacy and safety of SV for HF-AMI are limited to the Chinese patient population.

There are several limitations existed in this meta-analysis. First, all patients are from China, which might affect the generality of our findings. Second, five included studies are with relatively low quality, modified Jadad Scale 3. Third, ACEI/ARB drugs in the control group varies, which might cause some bias. Four, significant heterogeneity existed during the analysis of some outcomes and subgroup analysis failed to explain the main sources of heterogeneity. Five, due to the lack of original data, we are unable to conduct more subgroup analysis based on other important parameters such as the age, and dose and course of SV. Six, some included studies did not report baseline values of efficacy endpoints, thus we compared the post-treatment values between the SV and ACEI/ARB groups instead of the changes of efficacy endpoints, which might.

## 5 Conclusion

For the treatment of HF-AMI, SV is more effective and safer than ACEI/ARB based on current evidence. However, more high-quality RCTs are still needed to verify above findings due to the low-quality of some included studies.

## Data Availability

The original contributions presented in the study are included in the article/Supplementary Material, further inquiries can be directed to the corresponding author.
