# Supplementary material for: The efficacy and safety of sacubitril/valsartan compared with ACEI/ARB in the treatment of heart failure following acute myocardial infarction: a systematic review and meta-analysis of randomized controlled trials
Source: Front Pharmacol. 2023 Aug 4;14:1237210. doi: 10.3389/fphar.2023.1237210 (PMC10436296; doi:10.3389/fphar.2023.1237210)

Supplementary figure 2A. Begg's funnel plot of left ventricular ejection fraction.

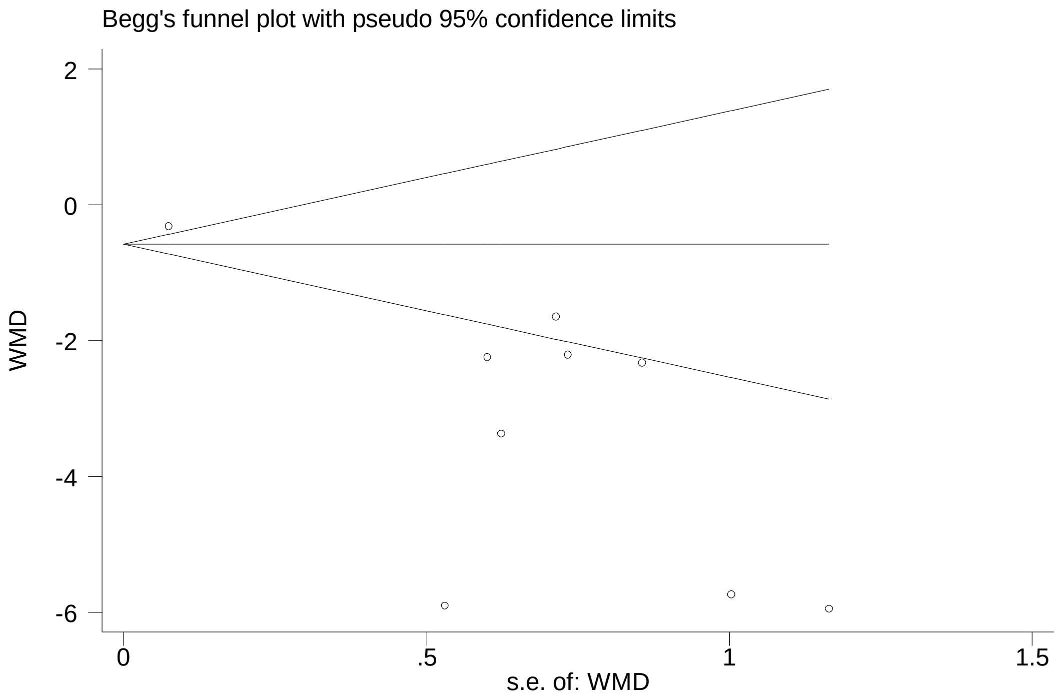

Supplementary figure 2B. Begg's funnel plot of NT-proBNP N-terminal pro-B type natriuretic peptide.

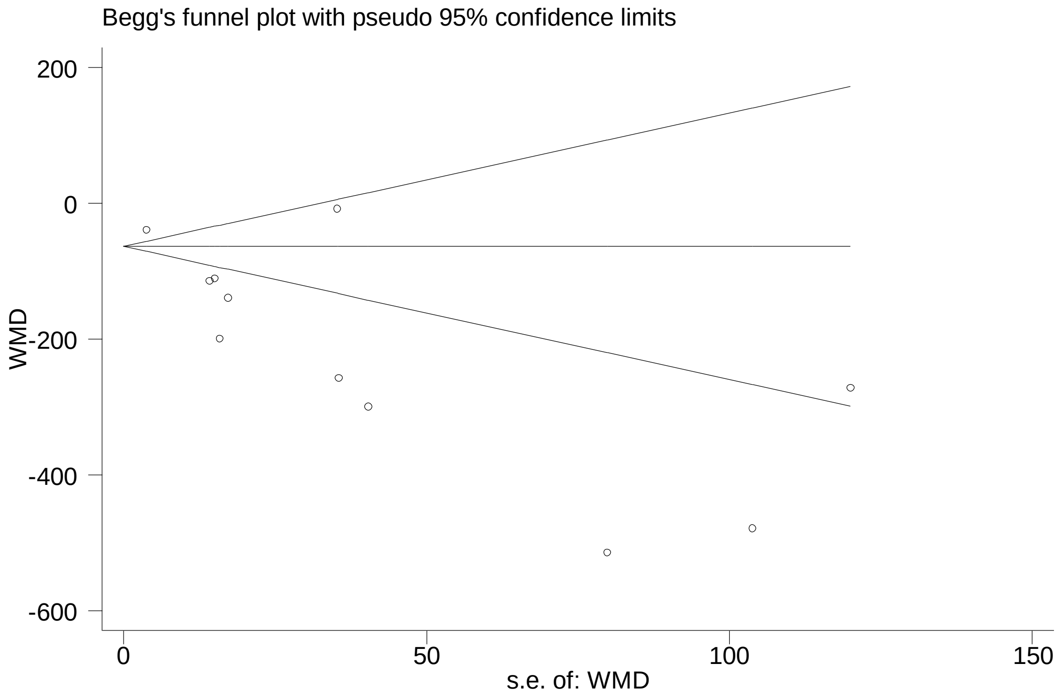

Supplementary figure 2C. Begg's funnel plot of left ventricular end-diastolic diameter.

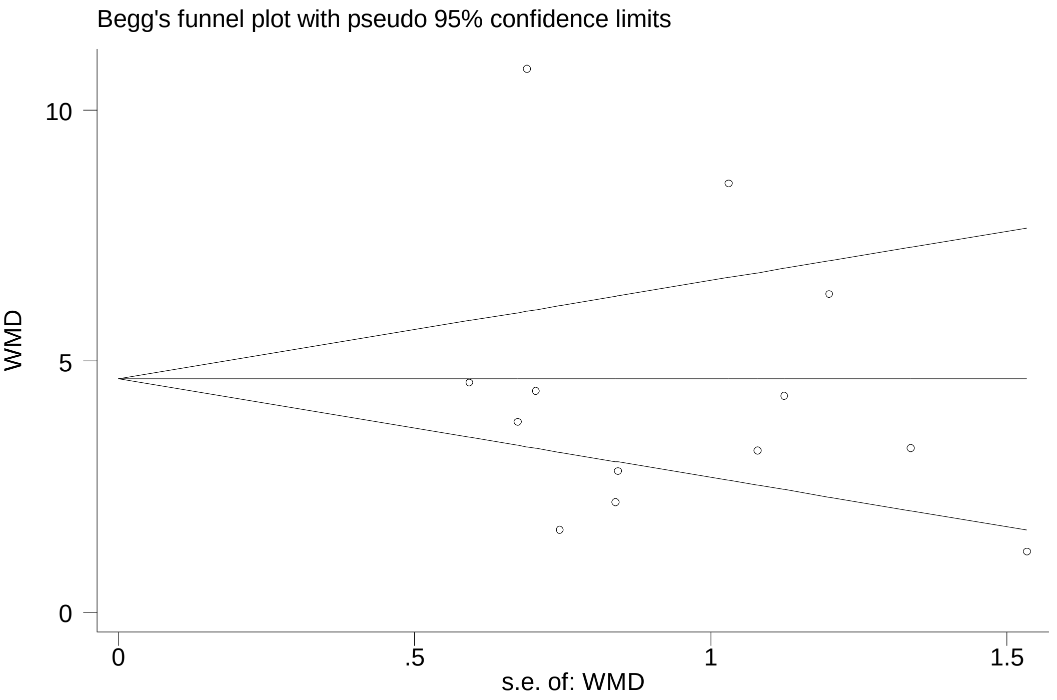

Supplement: Supplementary file 2 [file Presentation2.PDF]
